# Correlation Between Self‐Efficacy and Problem‐Solving Behavioral Intentions Regarding Near Misses Among Nursing Interns: The Mediating Role of Psychological Safety

**DOI:** 10.1155/jonm/6743629

**Published:** 2026-10-05

**Authors:** Yuning Chen, Huijuan Lei, Xu Huang, Jiale Li, Wenli Liang, Yiling Yang

**Affiliations:** ^1^ Department of Nursing Guangdong Pharmaceutical University Guangzhou Guangdong China gdpu.edu.cn; ^2^ School of Health and Nursing Guangzhou Huali College Jiangmen Guangdong China

**Keywords:** mediating effect, near miss, nursing interns, psychological safety, self-efficacy

## Abstract

**Background:**

A near miss is an error or event that is detected or corrected before reaching or harming the patient. Near‐miss events among nursing interns may compromise patient safety and reflect challenges in clinical practice. Self‐efficacy and psychological safety are important psychological factors that may influence problem‐solving behavioral intentions regarding near misses. However, evidence on the relationship between these variables among nursing interns remains limited. This study aimed to explore the relationship between self‐efficacy and problem‐solving behavioral intentions regarding near misses and to examine the mediating role of psychological safety.

**Methods:**

This cross‐sectional survey was conducted among 556 nursing interns at the First Affiliated Hospital of Guangdong Pharmaceutical University from January 3, 2026, to March 3, 2026, yielding 532 valid responses. Validated scales were used to assess self‐efficacy, problem‐solving behavioral intentions regarding near misses, and psychological safety. Data were analyzed using SPSS 26.0 and AMOS 24.0.

**Results:**

The results indicated that nursing interns reported a moderate level of self‐efficacy, while their total scores for problem‐solving behavioral intentions regarding near misses and for psychological safety were both at a high level. Self‐efficacy was positively correlated with both problem‐solving behavioral intentions regarding near misses (*r* = 0.253, *p* < 0.001) and psychological safety (*r* = 0.301, *p* < 0.001), while psychological safety was positively correlated with problem‐solving behavioral intentions regarding near misses (*r* = 0.217, *p* < 0.001). Mediation analysis indicated that psychological safety statistically accounted for 20.5% of the total association between self‐efficacy and problem‐solving behavioral intentions regarding near misses.

**Conclusions:**

Self‐efficacy was positively associated with nursing interns’ problem‐solving behavioral intentions regarding near misses, and psychological safety statistically accounted for part of this association. Scenario‐based training, nonpunitive feedback, accessible reporting channels, and timely feedback on corrective actions may support interns in identifying, reporting, and reviewing near misses.

## 1. Introduction

Patient safety is a central component of healthcare quality management, and near misses provide important information for identifying potential risks in clinical practice [1, 2]. A “near miss” is generally defined as an event in which an error occurs and creates potential risk, but is detected, intercepted, or corrected before causing patient harm [3]. Dennison et al. [4] described a near‐miss medication incident in which a fourth‐year nursing student selected the wrong intravenous bag because it had been stored incorrectly; the error was intercepted before patient harm occurred [4]. Although near misses do not cause direct harm, they can reveal weaknesses in clinical processes, risk recognition, staff communication, and system‐level safeguards [5]. In a multicenter study of 1173 nursing students undertaking clinical placements in China, near misses accounted for 87.01% of the nursing adverse events reported by students [6]. Near misses are therefore not a peripheral issue in clinical placements but an important point of entry for identifying how interns respond to risk and where clinical education may require support.

Nursing interns participate in frontline patient care and may play an important role in identifying, intercepting, and managing near misses [7]. However, their clinical experience, risk recognition ability, and problem‐solving skills are still developing [8]. Previous studies of nursing students’ near‐miss‐related behaviors have mainly examined incident experiences and immediate handling, incident disclosure or reporting, and speaking up for patient safety. This literature indicates that student status, preceptor evaluation, clinical hierarchy, and ward climate are associated with whether students raise safety concerns [9]. These studies help explain silence and underreporting but provide less information about whether interns intend to remain involved after an immediate correction. Problem‐solving behavioral intentions regarding near misses address this subsequent process through five dimensions: incident reporting, sharing with colleagues, cause investigation, practice change, and continuous improvement [10]. Compared with reporting alone, this construct more fully represents interns’ willingness to move from risk recognition to analysis and improvement.

Self‐efficacy is an important psychological factor that may influence such problem‐solving behavioral intentions regarding near misses [11]. It refers to an individual’s belief in their capability to complete specific tasks and affects motivation, effort, persistence, and behavioral choices in challenging situations [12]. In nursing education, task‐specific self‐efficacy has been associated with students’ patient safety competence, risk appraisal, and confidence in seeking support when clinical problems occur [13]. Nursing interns with higher self‐efficacy may therefore be more likely to regard a near miss as a manageable safety problem and to report, share, and analyze it, whereas those with lower self‐efficacy may avoid the situation or rely on others because they doubt their judgment [14]. However, previous studies have generally linked self‐efficacy to broad patient safety competence or a single safety response, leaving limited direct evidence regarding its association with all five dimensions of problem‐solving behavioral intentions regarding near misses.

Managing a near miss also involves interpersonal risk; capability beliefs alone may therefore be insufficient for strong problem‐solving intentions. Psychological safety refers to individuals’ perception that they can raise questions, express concerns, or report risks within a team without fear of punishment, humiliation, or rejection [15]. Studies of registered nurses have associated psychological safety with safety voice, silence, and intentions to report safety events [16]. Beyond these direct associations, Lee and Dahinten [17] tested psychological safety as a mediator and found that it mediated the associations between inclusive leadership and nurses’ voice behaviors and error reporting. Qualitative research with prelicensure nursing students has further shown that experiences of psychological safety during clinical rotations vary with interactions with preceptors and the clinical team and are reflected in students’ willingness to ask questions, acknowledge uncertainty, and raise concerns [18]. In addition, a study of nursing students found that psychological safety partially mediated the association between self‐confidence and learning flow [19]. Together, these studies indicate that psychological safety can serve as an explanatory mechanism linking individual or organizational factors with subsequent behaviors. However, existing mediation evidence comes mainly from leadership and voice studies among registered nurses or from simulation‐based learning among nursing students; it has not examined whether psychological safety links nursing interns’ self‐efficacy with their five‐dimensional problem‐solving intentions regarding near misses in clinical practice.

Social cognitive theory [20–22] and the psychological safety framework [23] provide a basis for this proposed mediating pathway. Social cognitive theory emphasizes reciprocal relations among personal cognition, environment, and behavior. In the present study, self‐efficacy represents interns’ judgments of their capability, psychological safety represents their perception of the clinical team environment, and problem‐solving intentions represent their readiness to take follow‐up action. Interns with higher self‐efficacy may be more willing to communicate, seek support, and clarify a concern [24], whereas greater psychological safety may be associated with fewer concerns about blame, humiliation, and negative evaluation, allowing capability beliefs to be more readily reflected in intentions to report, share, analyze, and improve practice [25]. Psychological safety may thus statistically account for part of the association between self‐efficacy and problem‐solving intentions, but this indirect association requires empirical examination among nursing interns.

Taken together, existing literature has separately documented silence, underreporting, and temporary handling of near misses among nursing students; associations between self‐efficacy and students’ safety competence; and associations between psychological safety and nurses’ safety voice or event reporting. These three streams of evidence remain fragmented. Empirical mediation studies that simultaneously examine self‐efficacy, psychological safety, and the five‐dimensional construct of problem‐solving behavioral intentions regarding near misses among nursing interns are still lacking. To address this gap, the present study aimed to examine the relationships among self‐efficacy, psychological safety, and problem‐solving behavioral intentions regarding near misses among nursing interns and to test the statistical mediating role of psychological safety. The study extends the outcome beyond incident reporting to five interconnected dimensions—reporting, colleague sharing, cause investigation, practice change, and continuous improvement—and examines problem‐solving intentions by integrating personal capability beliefs with perceptions of the clinical environment.

## 2. Methods

### 2.1. Sample and Sample Size

This cross‐sectional study recruited nursing interns from the First Affiliated Hospital of Guangdong Pharmaceutical University between January 3, 2026, and March 3, 2026. Following Kendall’s recommendation of 5–10 participants per item [26], 260–520 valid responses were required for the 52‐item survey. After allowing for a 10% rate of invalid responses, the recruitment target was 289–578 participants.

### 2.2. Inclusion and Exclusion Criteria

Participants were eligible if they were: (1) full‐time 4‐year undergraduate or graduate nursing students; (2) currently undergoing clinical internship during the study period; and (3) able to understand the study information and willing to participate voluntarily.

Participants were excluded if they (1) had discontinued or withdrawn from the internship before data collection; (2) had cognitive or communication difficulties that could affect questionnaire completion; or (3) had a serious illness that precluded participation.

### 2.3. Measure

#### 2.3.1. General Information Questionnaire

The researcher‐developed general information questionnaire comprised 11 items covering gender, age, educational level, personality type, internship duration, night‐shift experience, family residence, relationships with the father, mother, and clinical preceptors or colleagues, and reasons for choosing nursing. These variables were all included based on previous literature [6, 27–32]. These factors may be statistically associated with problem‐solving behavioral intentions regarding near misses in this study.

#### 2.3.2. General Self‐Efficacy Scale (GSES)

Self‐efficacy was assessed using the 10‐item GSES developed by Zhang and Schwarzer [33] and validated in a Chinese population by Wang et al. [34]. The unidimensional scale uses a 4‐point response format ranging from 1 (*not at all true*) to 4 (*exactly true*), with no reverse‐scored items. Item scores are summed to yield a total score of 10–40, with higher scores indicating greater self‐efficacy. Cronbach’s *α* was 0.940 in the present study.

#### 2.3.3. Instrument for Intention of Second‐Order Problem‐Solving

The conceptual framework of nurses’ intention for second‐order problem‐solving behavior regarding near misses and the corresponding assessment tool, proposed by Yang Yi [35], were used to assess participants’ behavioral intention to solve near‐miss problems. Problem‐solving behavioral intentions regarding near misses is defined as willingness or intention to investigate the root causes and seek preventive countermeasures in order to solve the problem [35]. The construct refers to an individual’s willingness to investigate underlying causes and pursue preventive actions after a near miss. Items are rated from 1 (*strongly disagree*) to 5 (*strongly agree*), with higher scores indicating stronger problem‐solving intentions. The instrument comprises five dimensions: incident reporting, sharing with colleagues, cause investigation, practice modification, and continuous improvement. Cronbach’s α was 0.914 in the present study. Incident reporting refers to reporting a near miss to their managers (e.g., the head nurse) and whether they are willing to register and file the event as required. Sharing with colleagues refers to whether nurses are willing to actively communicate and share relevant information with colleagues after a near miss, and whether they support their colleagues’ sharing behavior. Cause exploration refers to whether nurses are willing to systematically analyze the root causes from multiple aspects (individual, process, management, etc.) after a near miss, rather than staying at the surface level. Practice change refers to proposing or taking specific improvement measures after analyzing the causes, in order to prevent the event from happening again. Continuous improvement refers to whether nurses are willing to continuously track the effects of improvements and internalize the lessons learned from near misses into long‐term learning, so as to continuously improve the safety level.

#### 2.3.4. Psychological Safety Scale

Psychological safety was assessed using the eight‐item scale developed by Yu et al. [36], adapted from Anderson and West’s instrument. Items are rated on a 5‐point scale from 1 (*strongly disagree*) to 5 (*strongly agree*), yielding a total score of 8–40; higher scores indicate greater psychological safety. Cronbach’s *α* was 0.824 in the present study. In the Chinese version by Yu et al., standardized factor loadings exceeded 0.50 [37], and the average variance extracted was 0.518, which is above the 0.50 cutoff recommended by Fornell and Larcker [38].

### 2.4. Data Collection

The questionnaire was generated using an online survey platform (https://www.wjx.cn/), and a Quick Response (QR) code was created for WeChat (a Chinese instant messaging application). Subsequently, this QR code was distributed via WeChat to the participating nursing interns. Data analysis was conducted following the collection of all completed questionnaires. The survey targeted full‐time nursing interns engaged in clinical practicum at the First Affiliated Hospital of Guangdong Pharmaceutical University. All 556 interns who met the inclusion and exclusion criteria of the study agreed to participate and completed the questionnaire. After the data were collected, the questionnaires were checked for quality. Twenty‐four responses were excluded because the completion time was less than 90 s or because a repetitive response pattern was detected, leaving 532 valid questionnaires and a valid response rate of 95.7%.

### 2.5. Quality Control

To ensure research consistency, the questionnaire included standardized instructions detailing the study’s objectives, significance, and principles of confidentiality. Data collection was administered by professionally trained independent investigators unaffiliated with the research team, who used a standardized script to explain participants’ rights and ensured anonymity and voluntary participation in the absence of researchers or instructors. The survey system was configured to allow only one submission per IP address and device to prevent duplicate responses. Before the questionnaire officially began, the informed consent form was displayed. Participation and completion of the questionnaire were taken as consent. All items were mandatory, and the platform prompted participants to complete unanswered items, thereby minimizing missing data. Responses were considered invalid if the completion time was less than 90 s or if the same response option was selected for more than 90% of the scale items.

### 2.6. Data Analysis

Data analysis for this study was performed using SPSS 26.0 and AMOS 24.0 software. The significance level for all statistical tests was set at *α* = 0.05, with a *p*‐value < 0.05 considered statistically significant. Categorical variables (e.g., gender, education level, personality, internship duration, night‐shift experience, teacher–student relationship, family residence, paternal relationship, maternal relationship, and reasons for choosing nursing) were described using frequencies (*n*) and percentages (%), while continuous variables (e.g., age, self‐efficacy, near misses, and psychological safety scores) were presented as mean ± standard deviation (SD). Depending on the normality of the data distribution, Pearson bivariate correlation analyses were conducted to evaluate the relationships among nursing interns’ self‐efficacy, problem‐solving behavioral intentions regarding near misses, and psychological safety. We ran a multiple linear regression to identify the independent predictors of near‐miss problem‐solving intentions. Next, we used AMOS 24.0 to build a structural equation model linking self‐efficacy, psychological safety, and near‐miss problem‐solving intentions. Item parceling was applied to the unidimensional self‐efficacy and psychological safety scales following Bandalos [39] and Little et al. [39, 40]. An acceptable model fit was indicated by *χ* ^2^/df < 5, comparative fit index (CFI) and Tucker–Lewis index (TLI) > 0.90, and root mean square error of approximation (RMSEA) < 0.08. The indirect effect was evaluated using 5000 bootstrap resamples and was considered statistically significant when its 95% confidence interval (CI) did not include zero. Model fit was assessed using *χ* ^2^/df, the RMSEA, the CFI, and the TLI. Values of *χ* ^2^/df < 5, CFI and TLI > 0.90, and RMSEA < 0.08 were considered acceptable.

### 2.7. Ethical Considerations

This study was conducted in accordance with the Declaration of Helsinki and was approved by the Ethics Committee of the First Affiliated Hospital of Guangdong Pharmaceutical University (Approval No. 2026IIT‐32). The informed consent form and instructions for completion were again presented on the first page of the electronic questionnaire. Potential participants received study information through the online platform before accessing the questionnaire. Participation was voluntary, and questionnaire submission was taken to indicate informed consent.

## 3. Results

### 3.1. Participants’ Sociodemographic Characteristics

This study included a total of 532 valid questionnaires. Participants had a mean age of 22.08 years (SD = 1.73); 447 (84.0%) were aged 20–24 years. Of the 532 participants, 274 (51.5%) were male and 258 (48.5%) were female. Most held a bachelor’s degree (*n* = 465, 87.4%), had completed more than 3 months of internship (*n* = 338, 63.5%), and had night‐shift experience (*n* = 475, 89.3%). In terms of personality types, 162 participants (30.5%) identified as Type A (impatient, competitive, and energetic), 217 (40.8%) as Type B (relaxed, easy‐going, and steady), 37 (7.0%) as Type C (suppressive, conflict‐avoidant), and 116 (21.8%) as Type D (anxious, irritable, and socially withdrawn). Furthermore, 58.5% of the nursing interns reported their relationships with mentors or colleagues as “good” or better. The majority of nursing interns (90.6%) resided in urban areas. In terms of family dynamics, 72.4% and 72.6% of participants reported having a “good” or better relationship with their fathers and mothers, respectively. Finally, 70.0% of the participants indicated they chose the nursing profession for personal reasons. Detailed data are presented in Table 1.

**TABLE 1:** Sociodemographic characteristics of participants (*n* = 532).

Demographics	Mean (SD)	*n* (%)
Gender		
Male		274 (51.5)
Female		258 (48.5)
Age (years)	22.08 ± 1.73	
< 20		36 (6.8)
20–24		447 (84)
> 24		49 (9.2)
Education level		
Bachelor’s degree		465 (87.4)
Master’s degree or higher		67 (12.6)
Personality		
Type A		162 (30.5)
Type B		217 (40.8)
Type C		37 (7.0)
Type D		116 (21.8)
Internship duration (months)		
≤ 3		194 (36.5)
4–6		320 (60.2)
> 6		18 (3.4)
Night‐shift experience		
Yes		475 (89.3)
No		57 (10.7)
Relationship with clinical preceptors or colleagues	3.57 ± 1.26	
Very poor		41 (7.7)
Poor		77 (14.4)
Average		103 (19.4)
Good		158 (29.7)
Very good		153 (28.8)
Place of family residence		
Urban		482 (90.6)
Rural		50 (9.3)
Relationship with father	4.05 ± 1.12	
Very poor		16 (3.0)
Poor		37 (7.0)
Average		94 (17.7)
Good		143 (26.9)
Very good		242 (45.5)
Relationship with mother	4.03 ± 1.05	
Very poor		10 (1.9)
Poor		44 (8.3)
Average		92 (17.3)
Good		158 (29.7)
Very good		228 (42.9)
Reason for choosing a nursing major		
Personal choice		372 (69.9)
Family’s choice		137 (25.8)
Admission adjustment		13 (2.4)
Other reasons		10 (1.9)

### 3.2. Common Method Bias Test

We performed Harman’s single‐factor test using exploratory factor analysis. All items from the three scales—self‐efficacy, psychological safety, and problem‐solving behavioral intentions regarding near misses—were entered together into an unrotated principal component analysis. The first factor accounted for 21.65% of the total variance, below the prespecified 40% threshold, suggesting that substantial common method bias was not detected by Harman’s single‐factor test [41, 42].

### 3.3. Levels and Dimensional Scores of Self‐Efficacy, Problem‐Solving Behavioral Intentions Regarding Near Misses, and Psychological Safety

The total score for each scale is the sum of the raw scores of its items. The minimum values for each dimension came from different individuals, so simply adding them up does not necessarily equal the minimum value of the total scale in the sample. For descriptive interpretation, each theoretical score range was divided into three equal‐width intervals. GSES scores of 10.00–20.00, 20.01–30.00, and 30.01–40.00 were classified as low, moderate, and high, respectively. Using the same approach, scores of 23.00–53.67, 53.68–84.33, and 84.34–115.00 represented low, moderate, and high problem‐solving behavioral intentions regarding near misses, respectively. Based on these descriptive categories, self‐efficacy was moderate, whereas problem‐solving behavioral intentions regarding near misses and psychological safety were high. Full details are shown in Table 2.

**TABLE 2:** Levels and dimension scores of self‐efficacy, problem‐solving behavioral intentions regarding near misses, and psychological safety (*n* = 532).

Variables	Items	Observed range	Score (*X̄* ± SD)	Mean item score (*X̄* ± SD)
Self‐efficacy	10	12–39	27.36 ± 5.58	2.74 ± 0.56
Problem‐solving behavioral intentions regarding near misses	23	34–115	86.45 ± 15.40	3.76 ± 0.67
Event reporting	3	4–15	11.55 ± 2.52	3.85 ± 0.84
Sharing with colleagues	4	5–20	13.52 ± 3.42	3.38 ± 0.86
Cause investigation	7	7–35	26.05 ± 5.32	3.72 ± 0.76
Practice modification	5	6–25	19.20 ± 3.74	3.84 ± 0.75
Continuous improvement	4	5–20	16.14 ± 2.89	4.03 ± 0.72
Psychological safety	8	10–40	29.71 ± 6.22	3.71 ± 0.78

*Note: X̄* = mean; low, moderate, and high levels were defined descriptively by dividing each scale’s theoretical score range into three equal‐width intervals; these categories are not validated clinical cutoffs.

Abbreviation: SD = standard deviation.

### 3.4. The Relationships Among Self‐Efficacy, Psychological Safety, and the Dimensions of Problem‐Solving Behavioral Intentions Regarding Near Misses

As shown in Table 3, self‐efficacy was positively correlated with overall problem‐solving intentions and all five dimensions (*r* = 0.197–0.253, all *p* < 0.01), with the strongest correlation observed for sharing with colleagues (*r* = 0.251). Self‐efficacy was positively correlated with psychological safety (*r* = 0.301, *p* < 0.001), and psychological safety was positively correlated with problem‐solving intentions regarding near misses (*r* = 0.217, *p* < 0.001).

**TABLE 3:** Relationship between self‐efficacy and the dimensions of problem‐solving behavioral intentions regarding near misses (*n* = 532).

	1	2	3	4	5	6	7	8
1. Problem‐solving behavioral intentions regarding near misses	1							
2. Event reporting	0.785^∗∗^	1						
3. Sharing with colleagues	0.845^∗∗^	0.596^∗∗^	1					
4. Cause investigation	0.912^∗∗^	0.652^∗∗^	0.691^∗∗^	1				
5. Practice modification	0.880^∗∗^	0.645^∗∗^	0.677^∗∗^	0.734^∗∗^	1			
6. Continuous improvement	0.826^∗∗^	0.571^∗∗^	0.650^∗∗^	0.683^∗∗^	0.678^∗∗^	1		
7. Self‐efficacy	0.253^∗∗^	0.208^∗∗^	0.251^∗∗^	0.226^∗∗^	0.197^∗∗^	0.200^∗∗^	1	
8. Psychological safety	0.217^∗∗^	0.182^∗∗^	0.225^∗∗^	0.182^∗∗^	0.181^∗∗^	0.162^∗∗^	0.301^∗∗^	1

^∗∗^ *p* < 0.01.

### 3.5. Multiple Linear Regression Analysis of Factors Influencing Problem‐Solving Behavioral Intentions Regarding Near Misses

As shown in Table 4, before multiple linear regression, binary variables (gender, educational level, night‐shift experience, and family residence) were coded as 0 or 1 using the reference categories, as shown in Table 4. Age was entered as a continuous covariate. Personality type, internship duration, and reason for choosing nursing were represented by *k* − 1 dummy variables. Relationships with clinical preceptors or colleagues, fathers, and mothers were entered as ordinal covariates scored from 1 (*very poor*) to 5 (*very good*).

**TABLE 4:** Variable coding scheme.

Variable	Coding
Gender	0 = male as reference, 1 = female
Age (years)	Treated as a continuous covariate (actual age in years)
Education level	0 = bachelor’s degree as reference, 1 = master’s degree or higher
Personality	Dummy coded (Type B as reference): Type A (1 = Type A; 0 = others), Type C (1 = Type C; 0 = others), Type D (1 = Type D; 0 = others)
Internship duration (months)	Dummy coded (4–6 months as reference): Short duration (1 = < 4 months, 0 = others), long duration (1 = > 6 months, 0 = others)
Night‐shift experience	0 = yes as reference, 1 = no
Relationship with clinical preceptors or colleagues	Treated as an ordinal continuous covariate: Ranked 1 (very poor) to 5 (very good)
Place of family residence	0 = urban as reference, 1 = rural
Relationship with father	Treated as an ordinal continuous covariate: Ranked 1 (very poor) to 5 (very good)
Relationship with mother	Treated as an ordinal continuous covariate: Ranked 1 (very poor) to 5 (very good)
Reason for choosing a nursing major	Dummy coded (personal choice as reference): Family’s choice (1 = family’s choice; 0 = others), admission adjustment (1 = admission adjustment; 0 = others), other reasons (1 = other reasons; 0 = others)

As shown in Table 5, all variance inflation factor (VIF) values were below 2, indicating no multicollinearity issues among the variables. In the multivariable model, gender, age, educational level, personality type, internship duration, night‐shift experience, family residence, relationships with clinical preceptors or colleagues, and reasons for choosing nursing were not significantly associated with problem‐solving intentions. Relationships with the father and mother, self‐efficacy, and psychological safety were positively associated with the outcome (all *p* < 0.05).

**TABLE 5:** Multiple linear regression analysis of factors influencing problem‐solving behavioral intentions regarding near misses (*n* = 532).

	Unstandardized coefficient		*β*	*t*	*p*	Collinearity statistics	
	*B*	SE				Tolerance	VIF
Constant	55.113	9.887	—	5.574	< 0.001	—	—
Gender	0.600	1.334	0.019	0.450	0.653	0.896	1.116
Age	−0.047	0.370	−0.005	−0.127	0.899	0.976	1.025
Education level	−2.201	2.166	−0.047	−1.106	0.310	0.771	1.298
Personality Type B	—	—	—	—	Reference	—	—
Personality Type A	−1.469	1.544	−0.047	−0.951	0.342	0.692	1.446
Personality Type C	−0.709	2.863	−0.012	−0.248	0.805	0.751	1.332
Personality Type D	1.401	1.862	0.038	0.753	0.452	0.674	1.485
Internship duration: 4–6 months	—	—	—	—	Reference	—	—
Internship duration: < 4 months	0.563	1.427	0.018	0.394	0.694	0.843	1.186
Internship duration: > 6 months	−0.061	4.075	−0.001	−0.015	0.988	0.733	1.363
Night‐shift experience	−1.735	2.230	−0.035	−0.778	0.437	0.837	1.195
Relationship with clinical preceptors or colleagues	−0.662	0.657	−0.054	−1.007	0.314	0.586	1.705
Place of family residence	−0.148	2.462	−0.003	−0.060	0.952	0.771	1.297
Relationship with father	2.066	0.769	0.146	2.685	< 0.010	0.571	1.752
Relationship with mother	1.897	0.759	0.129	2.499	< 0.050	0.628	1.593
Reason for choosing a nursing major: Personal choice	—	—	—	—	Reference	—	—
Reason for choosing a nursing major: Family’s choice	−1.837	1.499	−0.052	−1.225	0.221	0.926	1.079
Reason for choosing a nursing major: Admission adjustment	3.803	4.505	0.038	0.844	0.399	0.823	1.215
Reason for choosing a nursing major: Other reasons	8.479	5.208	0.075	1.628	0.104	0.796	1.256
Self‐efficacy	0.480	0.121	0.174	3.954	< 0.001	0.870	1.149
Psychological safety	0.312	0.110	0.126	2.834	< 0.010	0.854	1.172

*Note: B* = unstandardized regression coefficient; *β = *standardized regression coefficient; *p* *=* *p*‐value. *R* ^2^ = 0.138, *F* = 4.829.

Abbreviations: SE = standard error, VIF = variance inflation factor.

### 3.6. Mediating Role of Psychological Safety in the Association Between Self‐Efficacy and Problem‐Solving Behavioral Intentions Regarding Near Misses

Prior to analysis, all continuous scale scores were standardized using before analysis. The mediation model linking self‐efficacy to problem‐solving behavioral intentions regarding near misses is shown in Figure 1. The model showed acceptable fit (*χ* ^2^/df = 1.232, CFI = 0.995, TLI = 0.994, RMSEA = 0.021). The mother–child relationship was positively associated with self‐efficacy (*β* = 0.16, *p* = 0.007), whereas the father–child relationship (*β* = 0.02, *p* = 0.659) and the relationship with clinical preceptors or colleagues (*β* = 0.03, *p* = 0.759) were not. Self‐efficacy was positively associated with psychological safety (*β* = 0.38, *p* < 0.001), and psychological safety was positively associated with problem‐solving intentions (*β* = 0.16, *p* < 0.01). The direct association between self‐efficacy and problem‐solving intentions also remained significant (*β* = 0.24, *p* < 0.001).

**FIGURE 1:**
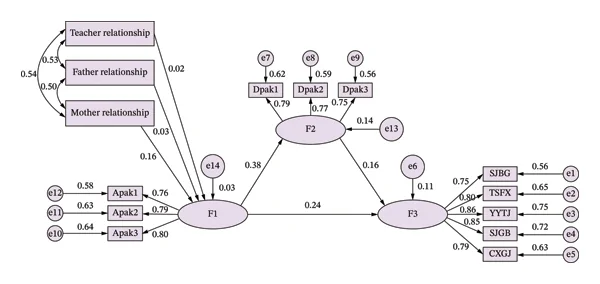
Structural equation model of the mediating role of psychological safety in the association between self‐efficacy and problem‐solving behavioral intentions regarding near misses (standardized estimates). Note. Ovals represent latent variables, and rectangles represent observed variables; F1 = self‐efficacy; F2 = psychological safety; F3 = problem‐solving behavioral intentions regarding near misses; SJBG = event reporting; TSFX = sharing with colleagues; YYTJ = cause investigation; SJGB = practice modification; CXGJ = continuous improvement; Apak1–Apak3 and Dpak1–Dpak3 represent item parcels for self‐efficacy and psychological safety, respectively. Double‐headed arrows indicate covariances among exogenous variables. e1–e12 denote measurement errors, except e6; e6 and e13 denote residual variances for F3 and F2, respectively. Path coefficients are standardized estimates.

## 4. Discussion

This study found that self‐efficacy was positively associated with nursing interns’ problem‐solving behavioral intentions regarding near misses, and that psychological safety statistically accounted for part of this association. Similar patterns have been reported among clinical nurses [17, 43], whereas the present study extends this evidence to nursing interns. Nursing interns are in a transitional stage of professional socialization and clinical role development [44]. At this stage, their responses to near misses may reflect not only confidence in handling clinical problems but also concerns about blame, evaluation, and other adverse consequences [45]. Psychological safety may therefore help explain why similar levels of self‐efficacy do not always correspond to equally strong problem‐solving intentions.

### 4.1. Levels of Self‐Efficacy, Problem‐Solving Behavioral Intentions Regarding Near Misses, and Psychological Safety

Nursing interns reported a moderate level of self‐efficacy (mean total score = 27.36, SD = 5.58), similar to the score reported by Fan et al. [46] (mean = 27.58, SD = 4.20) [46]. Clinical placement is an important transition in which nursing students apply theoretical knowledge to patient care and is often described as a period of transition shock [47]. In Bandura’s theory, self‐efficacy develops mainly through mastery experiences [14]. Although nursing interns have acquired theoretical knowledge, their opportunities to work independently and successfully manage unexpected clinical problems remain limited. They may therefore have had insufficient experience on which to base confidence in risk management. Timely correction by preceptors is essential for patient safety; however, when correction is perceived as harsh or punitive, interns’ belief that they can manage the problem may be weakened [48]. When near misses occur, interns with low self‐efficacy may first try to avoid the situation [49] rather than solve the problem. In contrast, interns with high self‐efficacy may be more likely to appraise near misses as manageable challenges rather than threats [50].

Nursing interns also reported a high level of psychological safety, suggesting some willingness to speak up but not a consistent sense that questions or risks could be raised safely in the clinical environment. This finding is consistent with previous research [46]. Nursing interns had a higher mean score than clinical nurses in Dong et al. [51] (mean total score 14.77 ± 5.18) [51]; however, differences in samples and study settings should be considered when interpreting this comparison. Both studies used the same version of the scale. This difference may reflect that career stage and clinical work roles have some influence on individuals’ psychological safety. Whereas self‐efficacy concerns whether an intern feels capable of responding, psychological safety concerns whether speaking up will lead to adverse interpersonal consequences. Nursing interns usually occupy a lower position in the clinical hierarchy and remain under continuous evaluation by preceptors and senior nurses. Even when they recognize a near miss, they may worry that questioning or reporting it will be interpreted as incompetence or disobedience or will adversely affect their placement evaluation. Previous research indicates that staff in higher power positions are generally more willing to express their views [52], whereas reporting concerns to authority figures such as preceptors or head nurses may be particularly difficult for nursing interns [53]. Negative workplace experiences, including verbal or physical aggression, deliberate neglect, psychological aggression, and sexual harassment, may further reinforce concerns about blame, exclusion, and negative evaluation [48]. Thus, recognizing a risk and feeling able to manage it may not be sufficient for an intern to speak up or report it.

Nursing interns’ problem‐solving behavioral intentions regarding near misses were likewise high. However, this level was lower than that reported by Feng et al. among registered nurses [54]. In this study, the mean scores for nursing interns across the five dimensions of problem‐solving behavioral intention ranged from 3.38 to 4.03, with an overall scale mean of 3.76. In comparison, Feng et al., using the same instrument with registered nurses, reported dimension means between 4.15 and 4.23, and an overall mean of about 4.20. Interns scored lower than registered nurses on all five dimensions, with the most noticeable difference on the “sharing with colleagues” dimension. This may suggest that nursing interns do have some willingness to take action, but that this intention has not yet reached a strong or consistent level. At the same time, this may also be related to differences in career stage and role authority. Registered nurses generally have more clinical experience, a more established professional identity, clearer responsibility for near‐miss management, and greater autonomy. Nursing interns work under supervision and may be uncertain whether they are authorized to question a decision, report an event, or initiate follow‐up. Their intentions may therefore be associated with both individual capability beliefs and the clinical environment: limited independent experience may constrain confidence, while hierarchy and evaluation pressure may constrain speaking up, reporting, and participation in follow‐up. Previous studies have likewise reported low levels of near‐miss reporting among nursing interns and nurses [55, 56]. This finding warrants attention because adverse nursing events have been reported more frequently among nursing interns than among experienced nurses, with rates ranging from 17.8% to 38.3% [57]. Near misses are often transient and difficult to observe; if they are not reported and followed up promptly, they may remain undetected, and opportunities to intercept harm and learn from the event may be lost [58]. Although the mean item score indicated a high descriptive level, nursing interns scored lower than registered nurses assessed with the same instrument, particularly for sharing with colleagues. This relative difference, rather than the absolute classification itself, identifies an area that may benefit from additional support in clinical education.

### 4.2. Relationship Between Self‐Efficacy and Problem‐Solving Behavioral Intentions Regarding Near Misses

Self‐efficacy was positively associated with nursing interns’ problem‐solving behavioral intentions regarding near misses (Table 3). This finding is consistent with social cognitive theory, which proposes that self‐efficacy is related to how individuals appraise difficult tasks and choose their responses [14]. When facing complex or unfamiliar clinical risks, interns with higher self‐efficacy may be more likely to trust their judgment, analyze the problem, and seek help from preceptors or team members when needed; those with lower self‐efficacy may withdraw or avoid the situation because they doubt their judgment. Previous research has similarly linked stronger capability beliefs with more active responses in unexpected clinical situations [49]. Self‐efficacy may therefore provide an important psychological basis for translating risk recognition into problem‐solving intentions. Self‐efficacy was positively associated with all dimensions of near‐miss problem‐solving intentions, with the strongest correlation observed for colleague sharing (*r* = 0.251). Because nursing interns have limited clinical experience and authority to act independently, sharing a concern with colleagues allows them to verify their judgment, obtain professional advice, and avoid bearing responsibility alone. Interns with higher self‐efficacy may be more willing to voice their assessment and seek team support. Self‐efficacy was therefore associated not only with individual intentions but also with risk communication and experience sharing within the team.

Most demographic characteristics were not significantly associated with problem‐solving intentions in this sample. The mother–child relationship was positively associated with both problem‐solving behavioral intentions regarding near misses and self‐efficacy, suggesting a possible role for emotional support and acceptance. Nursing interns are generally young and have limited clinical experience. When facing procedural risks, workload, and complex interpersonal relationships, understanding and support from mothers may be associated with less anxiety and fear of failure, a more positive appraisal of personal capability, and greater willingness to acknowledge a problem and seek help. Previous research has likewise associated emotional or material parental support with lower anxiety and fear and higher self‐efficacy among nursing students [59]. The mother–child relationship may thus be directly associated with problem‐solving intentions and may also be linked to them through self‐efficacy.

Unlike the mother–child relationship, the father–child relationship was positively associated with problem‐solving behavioral intentions but was not significantly associated with self‐efficacy. Its relevance may therefore lie more in how nursing interns communicate with authority figures than in their beliefs about personal capability. In traditional Chinese family culture, fathers often occupy a rule‐setting and authority role [60]. Communication patterns formed through long‐term interactions with fathers may extend to interactions with clinical authority figures such as preceptors and head nurses. Previous research indicates that a positive father–child relationship is associated with constructive communication patterns [61]. Interns with positive experiences of communicating with authority may be more willing to ask questions, report risks, and seek support in hierarchical clinical settings; those without such experiences may be more inclined toward compliance, silence, or avoidance. The mother–child relationship may therefore reflect an emotional support–capability belief pathway, whereas the father–child relationship may reflect an authority communication–behavioral expression pathway. From a cross‐cultural perspective, in a systematic review, [62] found that in countries that emphasize individualistic cultures such as the United States, parents’ confidence in parenting tends to decline as their children grow, and their promotive parenting behaviors may decrease. In collectivist cultural contexts such as China, however, parental self‐efficacy does not decline but rather increases as children enter adolescence, and this change is positively associated with children’s behavior and mental health. These interpretations represent culturally informed hypotheses rather than mechanisms directly tested in the present cross‐sectional study.

Beyond these psychosocial factors, no statistically significant associations were found between problem‐solving behavioral intentions regarding near misses and gender, age, educational level, personality type, placement duration, night‐shift experience, relationships with preceptors or colleagues, family residence, or reasons for choosing nursing. The relative similarity of participants’ educational stage and clinical placement arrangements may partly account for this pattern. General demographic characteristics may therefore be insufficient to distinguish nursing interns’ problem‐solving intentions, whereas psychological state, capability beliefs, and family interaction patterns may be more informative. However, a nonsignificant finding does not establish the absence of an association and should be interpreted cautiously.

### 4.3. The Mediating Role of Psychological Safety

As shown in Table 6, psychological safety statistically accounted for 20.5% of the total association between self‐efficacy and problem‐solving behavioral intentions regarding near misses. Interns with higher self‐efficacy may have greater confidence in their clinical judgment and may be more likely to appraise uncertainty as a manageable challenge rather than an uncontrollable threat [63]. This capability belief may be accompanied by less concern about criticism from preceptors, rejection by colleagues, or adverse placement evaluations, allowing interns to express questions and alternative views more readily. This interpretation is consistent with previous research on the association between self‐efficacy and psychological safety [64].

**TABLE 6:** Analysis of total, direct, and indirect effects with psychological safety as the mediator (*n* = 532).

Effect type	Effect value	SE	95% CI		*p*	Proportion (%)
			LLCI	ULCI		
Total effect	0.298	0.049	0.199	0.390	< 0.001	—
Direct effect	0.237	0.056	0.127	0.346	< 0.001	79.5%
Indirect effect	0.061	0.023	0.019	0.109	< 0.010	20.5%

*Note:* Standardized effects are reported.

Abbreviations: CI = confidence interval, LLCI = lower limit of the confidence interval, SE = standard error, ULCI = upper limit of the confidence interval.

The statistical mediating role of psychological safety may be related to the interpersonal risks involved in managing near misses. Nursing interns have limited clinical experience and relatively little power or voice within the team. Raising a concern or reporting a near miss may be interpreted as exposing their own lack of competence or challenging the judgment of a preceptor or another health professional. Even when interns recognize a risk and trust their judgment, concerns about blame, exclusion, or an adverse placement evaluation may lead them to remain silent. Previous research similarly identifies fear of blame and interpersonal pressure as barriers to speaking up about safety among nurses and nursing students [65]. Greater psychological safety may lessen these concerns and make interns more willing to move from noticing a problem to reporting it, communicating with others, and participating in follow‐up. Whereas previous studies have focused mainly on safety voice or incident reporting, the present study further describes psychological safety as a possible bridge between capability beliefs and problem‐solving intentions regarding near misses.

In this cultural and organizational context, nursing interns must consider not only the clinical problem but also whether raising it could offend a preceptor, disrupt team relationships, or cause another person to lose face. Self‐efficacy concerns whether an intern feels capable of judging and managing the problem, whereas psychological safety concerns whether the intern feels able to voice the concern and act on it. Without psychological safety, even interns with strong capability beliefs may remain silent to avoid interpersonal conflict [66]. Conversely, when asking questions is not met with blame and alternative views are respected, self‐efficacy may be more readily reflected in problem‐solving intentions. Together, these findings are consistent with a possible pathway in which higher self‐efficacy is associated with greater psychological safety, and both are associated with stronger intentions to act on near misses. Because the data were cross‐sectional, the temporal ordering of these associations cannot be established.

## 5. Conclusion

This study found that self‐efficacy was positively associated with problem‐solving behavioral intentions regarding near misses among Chinese nursing interns, and that psychological safety statistically accounted for 20.5% of the total association between self‐efficacy and problem‐solving intentions. These findings suggest that both capability beliefs and perceptions of interpersonal safety within the clinical team are relevant to nursing interns’ intentions to manage near misses. Cultural interpretations concerning face, harmony, and authority should be regarded as hypotheses for future research rather than conclusions established by this study.

### 5.1. Implications

For clinical nursing education, nursing schools and clinical training sites could incorporate near‐miss management into preplacement orientation and rotation‐based teaching. Training should move beyond explaining reporting rules. Graded simulations based on common near‐miss scenarios, such as medication administration, patient identification, and handover, could require interns to complete the full sequence of identifying the risk, pausing the task, explaining the concern to the preceptor, verifying the issue, reporting it, and participating in a debrief. After simulations or actual events, preceptors could use a standard debrief form to assess risk recognition, escalation communication, and follow‐up and provide specific, nonhumiliating feedback. Brief anonymous assessments at the start of placement and midway through a rotation could be used to identify interns reporting low self‐efficacy or psychological safety, followed by one‐to‐one coaching and additional scenario practice when needed.

For hospital patient‐safety management, hospitals could add an intern‐accessible, simplified near‐miss reporting route to the existing incident‐reporting system. Policy should explicitly authorize interns to pause and request verification when uncertain and state that good‐faith reporting will not be tied directly to placement grades or punitive appraisal. Each unit should define the recipient, escalation sequence, and feedback timeframe. Once a report is received, immediate risk should be addressed first, followed by a nonpunitive review led by the preceptor and patient‐safety staff and focused on process and system factors. Intern‐reported events could be reviewed monthly to identify recurring scenarios, repeated hazards, and completion of corrective actions, with feedback showing what was reported, what was done, and what changed. Implementation could be monitored through feedback timeliness, action‐closure rates, and recurrence of hazards rather than report counts alone.

## 6. Limitations

This study used cross‐sectional data collected at one time point. Future research should use a longitudinal design, with data collected at the early, middle, and late stages of internship, to track changes over time. The single‐center convenience sample from one university‐affiliated tertiary hospital may reflect a local organizational safety culture and limits generalizability to nursing interns in other regions and hospital settings. Future multicenter studies with more geographically diverse samples are needed to assess the generalizability of these findings. In addition, approximately 80% of the variance in problem‐solving intentions remained unexplained. Factors such as punitive safety culture, workload, preceptor support, and the accessibility of reporting channels may account for additional variance.

## Author Contributions

Yuning Chen contributed to writing–original draft, data curation, formal analysis, software, and visualization.

Huijuan Lei contributed to writing–original draft, conceptualization, investigation, and methodology.

Xu Huang contributed to investigation, data curation, and validation.

Jiale Li contributed to supervision and writing–review and editing.

Wenli Liang contributed to supervision, project administration, and writing–review and editing.

Yiling Yang contributed to supervision, writing–review and editing, project administration, and funding acquisition.

## Funding

This work was supported by the School‐level Innovation Training Project of Guangdong Pharmaceutical University [Grant No. 202504302136]; the Guangdong Medical Research Fund [Grant No. A2024452]; and the Characteristic Innovation Project for General Universities of Guangdong Province [Grant No. 2023WTSCX040].

## Disclosure

All authors read and approved the final manuscript.

## Ethics Statement

This study was conducted in accordance with the Declaration of Helsinki and was approved by the Ethics Committee of the First Affiliated Hospital of Guangdong Pharmaceutical University (Approval No. 2026IIT‐32). Potential participants were briefly informed of the study details via an online platform prior to the investigation. Participation was strictly voluntary, and completion of the questionnaire was regarded as implied informed consent.

## Consent

The authors have nothing to report.

## Conflicts of Interest

The authors declare no conflicts of interest.

## Data Availability

The datasets generated and/or analyzed during the current study are not publicly available because they contain information that could compromise participant confidentiality, but deidentified data may be available from the corresponding author upon reasonable request, subject to ethical approval.
